# The use of artificial intelligence to improve mycetoma management

**DOI:** 10.1371/journal.pntd.0011914

**Published:** 2024-02-08

**Authors:** Hyam Omar Ali, Lamis Yahia Mohamed Elkheir, Ahmed Hassan Fahal

**Affiliations:** 1 Mycetoma Research Centre, University of Khartoum, Khartoum, Sudan; 2 The Faculty of Mathematical Sciences, University of Khartoum, Khartoum, Sudan; 3 The Faculty of Pharmacy, University of Khartoum, Khartoum, Sudan; OSWALDO CRUZ FOUNDATION, BRAZIL

## Background

Mycetoma is a serious, neglected tropical disease affecting the underprivileged populations in resource-constrained communities of the rural tropical and subtropical regions. It predominately affects individuals of low visibility and voice and continues to hinder and burden the poor, remote communities [1]. Furthermore, in endemic areas, the medical and health facilities are meagre and adequate treatment is lacking or inaccessible [2]. All these lead to a progressive disease with massive morbidities and serious medical, health, and socioeconomic consequences [3,4].

Mycetoma is a chronic disabling subcutaneous granulomatous inflammatory disease of fungal and bacterial origin [5,6]. The inflammatory granuloma progressively spreads to affect the skin, deep tissues, and bones, leading to massive tissue damage, destruction, and serious morbidities and can be fatal [6–8]. A disease characteristic is the triad of subcutaneous mass, multiple sinuses discharging purulent and sero-purulent discharge frequently containing grains [9,10].

The mycetoma development and the associated risk factors are still unclear. Likewise, the disease susceptibility, resistance, entry route, and incubation period are unclear [11–13]. Presently, the diagnosis of mycetoma is difficult and tedious [14]. The available diagnostic tests are invasive, time-consuming, of low sensitivity and specificity, and there is no point of care [15–17]. Furthermore, the available treatment of mycetoma is suboptimal, characterised by a low cure rate and high recurrence and follow-up rates, and the disease remains with patients for a while, if not for life [18,19].

Artificial intelligence (AI) continues evolving swiftly, with ongoing research and development in various domains. It can potentially bring about significant advancements in science, technology, and society. AI has made substantial inroads into the medical and healthcare arenas, revolutionising healthcare delivery, disease diagnosis, and management. Nowadays, there are many applications for AI in medical and health practice, to mention but a few: its use in medical imaging, disease diagnosis, drug discovery, personalised medicine, data predictive analytics, electronic health records, telemedicine and virtual health assistance, robotics surgery, drug dosage, treatment recommendations, health monitoring, mental health, drug adverse event detection and healthcare operations, and resource management [20,21].

Machine learning (ML) is a subset of AI that creates algorithms and models that enable computers to learn and make predictions or decisions based on data to improve performance without explicit programming. ML is essential for interpreting large datasets, automating tasks, and enhancing decision-making processes, applied across diverse domains like image and speech recognition, natural language processing, recommendation systems, autonomous vehicles, healthcare, and finance. The learning process involves adjusting model parameters based on input data through training on labelled examples. ML encompasses 3 main types: supervised learning, where models are trained on labelled datasets; unsupervised learning, which explores patterns in unllabeled data; and reinforcement learning, where agents learn by making decisions in an environment to maximise cumulative rewards [22,23].

Deep learning (DL) is a specialised field within ML that utilises artificial neural networks, particularly deep neural networks with multiple layers, to undertake intricate tasks. Mimic the human brain’s structure, these networks learn hierarchical representations of data, enabling them to capture complex patterns and features. Its key characteristic includes the reliance on neural networks, where connections between nodes are adjusted during the learning process. DL automatically extracts relevant features from raw data and reduces the need for manual feature engineering. Its applications are in AI span image and speech recognition, natural language processing, autonomous vehicles, healthcare, and game playing. DL can autonomously learn from vast datasets, making it well-suited for challenging tasks where traditional methods may struggle, though it often requires substantial computational resources for training [24,25]. Advances in hardware and software have facilitated its widespread adoption in various AI applications. These applications are valuable due to their potential to enhance the accuracy and efficiency of medical diagnostics, reduce human error, and improve patient treatment outcomes.

As the progress in mycetoma management, research and development has been sluggish and inadequate over the years, we thought the utility of AI might enhance and accelerate this progress. With this background, we believe the utility of AI can improve mycetoma patient management and research and development endeavours in several ways. Here are some aspects of this utility:

## Early case detection and diagnosis

Computer vision can assist in early and accurate disease detection through image analysis of mycetoma lesions by identifying key mycetoma visual characteristics, such as the presence of masses, grains, sinuses, and discharge. This ultimately leads to early case detection in field surveys and remote communities, eventually leading to early management and disease burden reduction [26]. Also, it can involve studying medical radiological images for bone changes for mycetoma diagnosis and patient follow-up.

Furthermore, the capacity to distinguish mycetoma from other skin conditions by recognising distinctive patterns and lesions can lead to prompt interventions. One study reported the utility of ML models in identifying black skin diseases [27]. This study used AI models to classify 5 different skin NTDs, including mycetoma, based on skin images [27]. These models can further enhance the differentiation of mycetoma from other non-NTD skin diseases.

Recently, at the Mycetoma Research Centre (MRC), University of Khartoum, Sudan, computer vision technology was introduced for diagnosing mycetoma. This involved analysing the histological appearance of mycetoma granulomas, leading to the precise identification of different mycetoma types with a notable level of predictive accuracy [28].

Different imaging techniques, including ultrasound, X-ray, CT scans, and MRI, can determine the disease spread along the tissue planes and its severity. A suggested MRI-based mycetoma grading system to determine the disease spread along the body plans and the disease severity was reported [29]. This grading system can benefit from enhancement by integrating an AI model to grade the disease precisely into early and advanced stages and predict the treatment modalities. Furthermore, this AI model can be used for other radiological imaging modalities.

## Treatment

AI is emerging as a powerful tool in medical imaging, biomarker discovery, genetics research, treatment planning, and patient monitoring [30,31]. In mycetoma, AI can predict the diagnosis, treatment plans, disease monitoring, progress, and treatment outcomes using different and variable datasets. These data can include different clinical, radiological, laboratory, histopathological, and cytological information and images.

Mycetoma is a deep-seated mycosis, and it is difficult to predict the disease deep spread clinically. Hence, computer vision can provide valuable information for adequate surgical intervention as it predicts the lesion boundaries and depth to ensure wide local excision during the surgical procedure [32].

In mycetoma, the treatment outcomes are unpredicted [2,33]. That depends on the disease type, site, spread, the patient’s immune status, and possibly a genetic background [34]. AI can analyse massive clinical data, genetic information, and lifestyle choices to predict potential drug interactions or contraindications, which is critical for patients on long-term medication and can support pharmacists in planning patients’ personalised treatment plans [35].

In general, AI-driven treatment predictive models are used to personalise patient treatment and predict the prognosis of many medical conditions [35]. ML algorithms were used to predict the risk of postoperative mycetoma recurrence after adequate medical and surgical treatment in patients seen at the MRC [36,37]. With the AI utility, mycetoma treatment failure and recurrence predictors can be identified early in the treatment course. Hence, the treatment plan can be modified to early surgical intervention to avoid unnecessary prolonged medical treatment to reduce the morbidity, drug side effects, cost, time waste, and detrimental consequences of mycetoma [35].

## Mycetoma epidemiology

Most of the mycetoma epidemiological characteristics are not well understood [3,4]. Likewise, its spatial geographical distribution and environmental occurrence predictors [38]. Also, the exact mechanism of mycetoma transmission remains unclear, although it is suspected that mycetoma may be transmitted through the traumatic introduction of environmental microorganisms into the subcutaneous tissue [2,4]. Moreover, the mapping of high-risk regions has not been fully achieved, all these hamper the development of effective prevention and control strategies.

Through computer vision, automated surveillance systems can identify potential mycetoma causative organisms’ localities and monitor patient density in these areas. Furthermore, computer vision can analyse satellite imagery and integrate it with other epidemiological data, offering a comprehensive perspective on mycetoma distribution. This approach can lead to the creation of epidemiological maps that aid in identifying endemic regions with suspected patients and planning interventions to reduce the disease prevalence and burden.

Different AI-driven approaches were used to predict the mycetoma distribution in Sudan [39,40]. Ecological niche modelling (ENM) was used to map the risk of mycetoma infection across Sudan and South Sudan, utilising scientific literature reported mycetoma cases, digital geographic information system (GIS), data layers of soil characteristics, land-surface temperature, and greenness indices [39]. Another study was conducted using machine learning to identify the environmental predictors of mycetoma in Sudan and to identify areas where these niche predictors are met by building a distribution model that utilised large demographic and clinical datasets of confirmed mycetoma patients seen at the MRC from 1991 to 2018 with a collection of environmental and water and hygiene-related datasets in a geostatistical framework [40]. The model successfully estimated the mycetoma burden in Sudan and produced estimates of the disease burden across the country. Thus, AI and ML can significantly assist in tracking the spread of mycetoma in different regions, identifying risk factors, and planning interventions to reduce the prevalence of the disease.

## Telemedicine and telehealth

In remote or underserved regions, computer vision has the potential to facilitate telemedicine initiatives by analysing images or videos submitted by patients, allowing for initial diagnosis and remote consultations [41]. Furthermore, telemedicine platforms can be enhanced with computer vision capabilities to grant access to clinicians, radiologists, histopathologists, dermatologists, and mycetoma specialists who can remotely diagnose cases and offer guidance on treatment. Patients can also employ smartphone images to communicate with healthcare providers, and AI can analyse these images to predict diagnoses and recommend treatment plans. Furthermore, AI can remotely monitor patients and improve patient treatment compliance.

The MRC has established peripheral satellite centres in villages where mycetoma is prevalent, aiming to manage and monitor patients within their communities to alleviate the socioeconomic burden [26]. MRC uses telemedicine technology to communicate with patients in these remote centres.

## Data analysis

Many centres and hospitals have massive clinical, laboratory, imaging, and epidemiological data. However, they are badly archived and unused, and AI can generate valuable information from such datasets [42–44]. AI can analyse the MRC massive data to predict clinical and treatment outcomes, improve diagnostic accuracy, and plan and improve treatment strategies. Epidemiological data analysis can support the designing and planning of mycetoma control and prevention measurements and programmes to reduce the disease burden and consequences [45–48].

## Healthcare management

Numerous tasks, including patient check-in and pharmacy operations, can be automated using AI-driven solutions and virtual assistants. These technologies enable a self-service approach to patient check-in, allowing patients to access their information and receive pertinent documents and instructions. Additionally, AI aids the statistical unit by streamlining information gathering, record updating, and analysing the available data [49].

The utilisation of robotic systems in pharmacy operations enhances the efficiency of medication management, as they assist in both medication dispensing and verification, thereby reducing the risk of errors and improving overall operational efficiency. AI methodologies not only automate these processes but also contribute to a superior patient experience by decreasing waiting time, minimising errors, and elevating the overall quality of care.

## Research and drug discovery

Despite the availability of some chemotherapeutic options for mycetoma patients, the cost and relative lack of efficacy has resulted in unsatisfactory progress in mycetoma prevention and treatment [3,4]. Advancements in drug discovery for mycetoma are quiescent, with no drugs currently advancing in the pipelines. This is due to the high costs required in the drug discovery and development process, for such diseases, usually cannot be redeemed by investors as mycetoma mainly affects poor communities who won’t eventually be able to pay for these drugs. Applying contemporary AI and ML methods to mycetoma drug discovery could offer a cost-effective and rapid alternative for mycetoma drug discovery. They provide powerful tools for screening large libraries of molecules for promising leads and for the rational design of new therapies [50,51]. AI and ML approaches could also be utilised in drug repurposing to rapidly and cheaply identify potentially useful drugs [52,53]. Pharmaceutical modelling and AI-guided drug discovery tools have successfully accelerated the identification of novel inhibitors and promising drug targets for several NTDs [54–56]. An interesting example is the development of the Robot Scientist “Eve,” a laboratory automation system that uses AI techniques to discover scientific knowledge through cycles of experimentation. Eve successfully repositioned several drugs against specific targets in parasites that cause tropical diseases, such as the anti-cancer compound TNP-470, which was found to be a potent inhibitor of dihydrofolate reductase of the malaria-causing *Plasmodium vivax* [57]. The success of these approaches could be replicated in the drug discovery efforts for mycetoma.

## Clinical trials

By several means, AI technologies can enhance clinical trials efficiency, accuracy, and effectiveness [58–62]. In mycetoma, clinical trials are lacking, the cause is multifactorial, and lack of experience and funds are important reasons. This AI can be instrumental in conducting that. It can help identify and recruit suitable participants using and analysing electronic health records, genetic data, and other patient information. AI algorithms can match eligible participants with trial criteria to accelerate recruitment and reduce time. Also, it can assist in optimising the clinical trial protocol design. AI algorithms can analyse historical data and predict the best trial design to increase the likelihood of obtaining meaningful results. Managing and analysing the vast data generated during a clinical trial is complex. AI can help in data management, organisation, analysis, and decision-making. Natural language processing can extract valuable information from clinical notes, reducing the need for manual data entry. It can predict patient outcomes and help identify potential safety concerns or adverse events early in a trial, leading to more proactive monitoring and faster decision-making. AI can help integrate real-world data, such as patient-generated data, wearable device data, and social media insights, into clinical trials. This can provide a more comprehensive understanding of the proposed study intervention. The technique can monitor patient compliance, enable real-time data collection during trials, and reduce the patients’ physical presence at the trial site. Furthermore, AI can assist in maintaining regulatory compliance by ensuring data accuracy, security, and privacy [58–62].

## Mycetoma genetic susceptibility

AI offers a wide array of models for investigating genetic susceptibility to diseases by analysing extensive genomic data to identify genetic factors that elevate the risk of disease occurrence [63].

Still, in mycetoma, the genetic background of the affected patients is unknown likewise, the disease susceptibility and resistance are unclear [34]. ML techniques are becoming increasingly vital in analysing genome-wide association (GWAS), next-generation sequencing (NGS) studies, and computational genetic mapping analysis [63,64]. Thus, such techniques could help to highlight new strategies to systematically interrogate the genome of patients and causative organisms to identify possible genetic risk factors and host–pathogen interactions in mycetoma. This, in turn, could greatly aid in early case detection and the design of more effective prevention strategies.

## Patient education

The mycetoma affected patients usually live with the disease and its consequences for life and continue to face different events and difficulties. AI-powered chatbots or virtual assistants can provide information and answer questions for these patients, helping them better understand their conditions and adhere to treatment plans and for community re-inclusion.

In conclusion, it is vital to note that while AI holds potential in managing mycetoma patients, it should always be used in conjunction with clinical judgement and expertise. It can support many epidemiological decisions and accelerate research and development activities. While AI can transform mycetoma healthcare positively, it raises important issues regarding data privacy, ethical considerations, regulatory compliance, and human oversight in medical decision-making [65,66]. Therefore, integrating AI into mycetoma care requires good planning and collaboration between medical and health professionals, data scientists, and policymakers to ensure its responsible and effective use.
